# Comparison between the recovery time of alfentanil and fentanyl in balanced propofol sedation for gastrointestinal and colonoscopy: a prospective, randomized study

**DOI:** 10.1186/1471-230X-12-164

**Published:** 2012-11-21

**Authors:** Wai-Meng Ho, Chia-Ming Yen, Chin-Hung Lan, Chung-Yi Lin, Su-Boon Yong, Kai-Lin Hwang, Ming-Chih Chou

**Affiliations:** 1 Institute of Medicine, Chung Shan Medical University, No. 110, Sec. 1, Jianguo N. Rd., Taichung, 402, Taiwan; 2 Department of Anesthesiology, Buddhist Tzu Chi General Hospital, Taichung Branch, No.66, Sec. 1, Fongsing Rd., Tanzih, Taichung, 427, Taiwan; 3 Department of Anesthesiology, School of Medicine, Tzu Chi University, No.701, Chung Yang Rd., Sec .3, Hualien, 970, Taiwan; 4 Department of Anesthesiology, Buddhist Tzu Chi General Hospital, No. 707, Sec. 3, Chung Yang Rd., Hualien, 970, Taiwan; 5 Division of Gastroenterology and hepatology, Buddhist Tzu Chi General Hospital, Taichung Branch, No.66, Sec. 1, Fongsing Rd., Tanzih, Taichung, 427, Taiwan; 6 Department of Pediatrics, Show Chwan Memorial Hospital, No.500, Sec 1, Chung Shan Rd., Changhua, 500, Taiwan; 7 Department of Public Health, Chung Shan Medical University, No. 110, Sec. 1, Jianguo N. Rd., Taichung, 402, Taiwan; 8 Division of Thoracic Surgery, Department of Surgery, Chung Shan Medical University Hospital, No. 110, Sec. 1, Jianguo N. Rd., Taichung, 402, Taiwan

**Keywords:** Balanced propofol sedation, Alfentanil, Fentanyl, Deep sedation, Diagnostic endoscopy, Cost benefit

## Abstract

**Background:**

There is increasing interest in balanced propofol sedation (BPS) titrated to moderate sedation (conscious sedation) for endoscopic procedures. However, few controlled studies on BPS targeted to deep sedation for diagnostic endoscopy were found. Alfentanil, a rapid and short-acting synthetic analog of fentanyl, appears to offer clinically significant advantages over fentanyl during outpatient anesthesia.

It is reasonable to hypothesize that low dose of alfentanil used in BPS might also result in more rapid recovery as compared with fentanyl.

**Methods:**

A prospective, randomized and double-blinded clinical trial of alfentanil, midazolam and propofol versus fentanyl, midazolam and propofol in 272 outpatients undergoing diagnostic esophagogastroduodenal endoscopy (EGD) and colonoscopy for health examination were enrolled. Randomization was achieved by using the computer-generated random sequence. Each combination regimen was titrated to deep sedation. The recovery time, patient satisfaction, safety and the efficacy and cost benefit between groups were compared.

**Results:**

260 participants were analyzed, 129 in alfentanil group and 131 in fentanyl group. There is no significant difference in sex, age, body weight, BMI and ASA distribution between two groups. Also, there is no significant difference in recovery time, satisfaction score from patients, propofol consumption, awake time from sedation, and sedation-related cardiopulmonary complications between two groups. Though deep sedation was targeted, all cardiopulmonary complications were minor and transient (10.8%, 28/260). No serious adverse events including the use of flumazenil, assisted ventilation, permanent injury or death, and temporary or permanent interruption of procedure were found in both groups. However, fentanyl is New Taiwan Dollar (NT$) 103 (approximate US$ 4) cheaper than alfentanil, leading to a significant difference in total cost between two groups.

**Conclusions:**

This randomized, double-blinded clinical trial showed that there is no significant difference in the recovery time, satisfaction score from patients, propofol consumption, awake time from sedation, and sedation-related cardiopulmonary complications between the two most common sedation regimens for EGD and colonoscopy in our hospital. However, fentanyl is NT$103 (US$ 4) cheaper than alfentanil in each case.

**Trial registration:**

Institutional Review Board of Buddhist Tzu Chi General Hospital (IRB097-18) and Chinese Clinical Trial Registry (ChiCTR-TRC-12002575)

## Background

Esophagogastroduodenal endoscopy (EGD) and colonoscopy are among the most widely utilized procedures worldwide that are performed with intravenous sedation, so effective sedation with prompt recovery is important. There is increasing interest in balanced propofol sedation (BPS) titrated to moderate sedation (conscious sedation) for endoscopic procedures [1-6].

BPS combines propofol with small doses of an opioid and a benzodiazepine. A positive synergistic interaction is noted among these drugs. Therefore, each drug’s therapeutic action is enhanced while the adverse effects of each are minimized because of the small doses used.

Compare with conventional sedation (a combination of a benzodiazepine and an opioid e.g. midazolam and meperidine), BPS can be used safely and effectively for diagnostic and therapeutic endoscopy and provides higher health care provider satisfaction and better patient cooperation.

Midazolam, fentanyl and alfentanil are the most preferred sedo-analgesics with short acting effects on γ-aminobutyric acid and μ-opiate receptors, which provide a rapid induction and recovery with minimal residual side effects for endoscopy [7,8].

Alfentanil, a rapid and short-acting synthetic analog of fentanyl, appears to offer some clinically significant advantages over fentanyl during outpatient anesthesia [9].

Alfentanil was also used as an adjuvant to midazolam for sedation of outpatients undergoing upper gastrointestinal endoscopy. The use of alfentanil resulted in improved operating conditions and a more rapid recovery as compared with patients sedated with midazolam alone. Patient acceptance was also higher [10].

Moreover, with its rapid recovery effect, alfentanil is superior to fentanyl in patient- controlled analgesia and sedation for colonoscopy [11].

Therefore, it is reasonable to hypothesize that low dose of alfentanil used in BPS might also be result in more rapid recovery as compared with fentanyl. However, there are few controlled studies with direct comparison between alfentanil and fentanyl in BPS.

In addition, deep sedation rather than moderate sedation for endoscopy was preferred by most of the patients undergoing diagnostic colonoscopy for health check-up in Taiwan [12,13].

Thus, we design this prospective, randomized, and double blind clinical trial to compare the effectiveness, the recovery and the safety profiles between small dose of alfentanil and fentanyl adjunct to BPS targeted to deep sedation for patients undergoing diagnostic EGD and colonoscopy for health examination. Cost benefit analysis between alfentanil and fentanyl might also be performed.

## Methods

### Patient selection and study design

This was a single-center, prospective, randomized, and double-blinded study of 260 consecutive patients undergoing diagnostic EGD and colonoscopy for health examination between July 2008 and June 2009. The study was conducted according to the Declaration of Helsinki and was approved by the Institutional Review Board of Tzu Chi General Hospital. Verbal and written informed consent for this study was obtained from each patient.

Clinical criteria for patient exclusion from BPS included patients of age less than 20 years; pregnancy; American Society of Anesthesiology (ASA) physical status class IV; history of allergies to propofol, soybeans, or eggs; chronic lung disease; history of drug or alcohol abuse; history of seizure disorder, sleep apnea, or difficult intubation; a short, thick neck or inability to open the mouth widely; history of complications with previous sedation and inability to provide informed consent.

**Figure 1:**
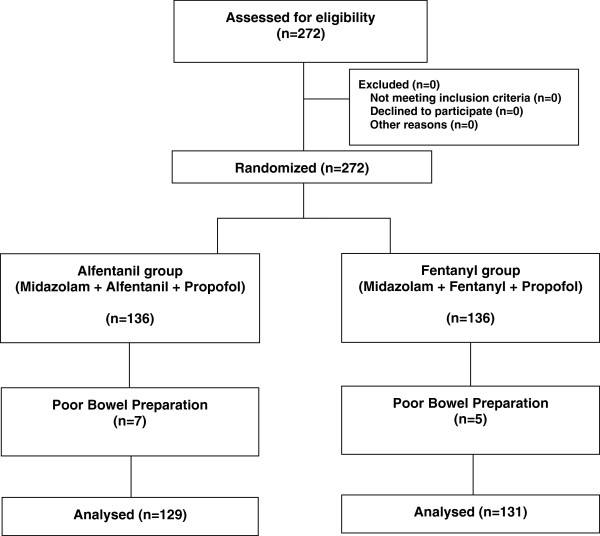
Flow diagram of the study.

A total of 272 patients were invited to participate. No patient was excluded for any of the exclusion criteria and all the screened patients consented to randomization. However, 12 patients were excluded eventually due to poor bowel preparation (7 in alfentanil group and 5 in fentanyl group). Thus, 260 patients completed the study (Figure 1).

All endoscopic procedures were performed by an experienced endoscopist who was faculty at the hospital. Patients were randomly assigned to 1 of 2 sedation protocols (alfentanil group [propofol in combination with midazolam and alfentanil] vs fentanyl group [propofol in combination with midazolam and fentanyl]) by using a computer-generated random sequence. Both randomization and the opioids used for analgesia were concealed from all patients, anesthesiologist, endoscopist, endoscopy nurses, recovery-room nurses, and an independent research nurse who responded for recovery evaluation in post-anesthesia room and 24-hour telephone follow-up survey.

### Sedation protocols

All sedatives and analgesics used for this study were administered by only one anesthesiologist. The opioid and midazolam were given by intravenous bolus injection at the initiation of sedation. All the patients received midazolam 2.5 mg, and in combined with alfentanil 0.25 mg (Rapifen® 1 mg/2 ml; Janssen-Cilag Limited, Belgium) for the alfentanil- propofol group or fentanyl 0.025 mg (Fentanyl-Fresenius 0.1 mg/2 ml; Bodene (Pty) Limited, South Africa) for the fentanyl-propofol group.

Thereafter, an initial bolus of propofol (0.5 mg/kg body weight) was given intravenously. Sedation was maintained with repeated doses of 10 to 20 mg propofol. The goal of sedation was deep sedation, based on ASA levels and the Observer's Assessment of Alertness/Sedation scale (OAAS scale) [14,15], which defines as a patient who responds purposely to a painful stimulus (e.g. trapezius squeeze) but fails to response to verbal or light tactile stimuli.

The gastroscopy was performed first and followed with colonoscopy. Repeated dose of alfentanil or fentanyl was administered just before the start of colonoscopy. Therefore, each patient’s total dose of alfentanil or fentanyl was 0.5 mg and 0.05 mg, respectively. During the insertion of the colonoscopy, deep sedation was also maintained with 10 to 20 mg increments of propofol. Usually no more propofol was added during the withdrawal phase.

All EGD and colonoscopy examinations in this study were performed by one experienced gastroenterologist using a standard video gastroscope (EG-590WR; Fujinon Corp., Saitama, Japan) and colonoscope (EC-450WL5; Fujinon Corp).

Blinding was achieved by having an independent research nurse, according to a grid known only to her, provided each patient a 1 ml tuberculin syringe containing either alfentanil 0.5 mg/ml or fentanyl 0.05 mg/ml.

Since the alfentanil and fentanyl were clear and colorless aqueous solutions, they were unable to be identified. Therefore, the anesthesiologist, gastroenterologist, nurses and patients were all blinded to treatment randomization.

### Patient monitoring

The baseline vital signs were recorded just before the procedure. Both preoxygenation with intranasal supplemental oxygen (6 L/min) and normal saline solution were provided to all patients at the initiation of the procedure. All patients were continuously monitored for heart rate, blood pressure, blood oxygen saturation (SpO2) by using an automated device (Datascope 3000; Datascope Corp., Montvale, N.J.) until full recovery. Respiratory effort, respiratory rate, and chest wall excursion were monitored by visual inspection and palpation. The anesthesiologist recorded all the monitoring parameters, total procedure time from the continuously running stopwatch and undertook chin lift, safety end points and advanced life-support.

Equipment for full resuscitation was available at all times within the endoscopy unit. Both flumazenil and naloxone could be used as reversal agents when severe adverse effects associated with the use of midazolam and opioids were suspected.

### Outcome measurements and definitions

The primary outcomes were to compare the recovery time and the procedure-related times (awake and total procedure times) between the two groups. Awake time was defined as the time interval between “colonoscope removal” and “patient reaching OAAS scale 5” which means that the patient responds appropriately to normal volume verbal cues by using either a verbal or physical response without delay or hesitation.

Total procedure time was defined as the time interval between “start of gastroscopy” and “colonoscope removal”. Recovery time was defined as the time interval between “colonoscope removal” and “discharge from the endoscopy unit”. If there was transient interruption of the procedure due to sedation-related adverse events, that time was subtracted from the total procedure time.

Secondary outcomes included the total amount of propofol used and the incidences of cardiopulmonary complications between the two groups. Cardiopulmonary complications included [1] systolic hypotension (systolic blood pressure < 90 mm Hg), [2] bradycardia (heart rate < 50 beats/min), [3] hypoxemia (pulse oximeter oxygen saturation (SpO2) < 90% on supplemental oxygen), and [4] permanent injury or death. If the SpO2 dropped to < 90% for >15 seconds, the patient’s chin was lifted. If the SpO2 dropped to < 85% for > 30 seconds, assisted ventilation was applied and an antagonist to midazolam (flumazenil) could be injected. The procedure was interrupted until normalization of the oxygen saturation. Transient interruption of the procedure was defined as procedure interruption due to sedation’s adverse events for > 30 seconds.

### Discharge evaluation and 24-hour follow-up

Upon completion of the EGD and colonoscopy procedure, patients were taken directly to the recovery area, where an initial set of pulse, blood pressure and pulse oximetry was taken immediately. An independent blinded research nurse determined the earliest time at which patients were judged to be fully alert, and time they were able to stand unassisted by the bed without hypotension (systolic blood pressure < 100 mmHg) or hypoxia (SpO2 <95% by pulse oximetry). When these points were reached, the patient was considered ready for discharge.

Before discharge, modified from Sipe et al. [15] and Cohn et al. [2], the patient was asked to fill out a short questionnaire using standard 10-cm visual analog scales to report the amount of pain they experienced during the procedure and overall satisfaction with the procedure. The patient was asked verbally whether they remembered being awake during the procedure.

Patients also were follow-up by phone 24 hours later and a follow-up questionnaire was completed (Appendix).

### Cost benefit analysis

Individual patient's anesthetic costs, including only sedative and analgesic consumed, would be calculated and analyzed between the groups.

### Statistical analysis

The t test was used for continuous data. The Chi square test or Fisher exact test was used for comparison of categorical data when appropriate. A P value of less than 0.05 was considered to be statistically significant. All data were analyzed by using the Statistical Package for the Social Sciences software, version 15.0 for Windows.

Calculation of sample size was based on the primary objective of the study, i.e., the recovery time of each sedation regimen. Based on our pilot study, the average recovery time of fentanyl sedation regimen was 15 minutes. We assumed that the difference of recovery times was around 2.5 to 3 minutes between the groups. Assuming a 10% dropout rate, 136 patients were needed in each group with an alpha value of .05 and a power of 80% [16].

## Results

### Study population and baseline characteristics

There were 272 patients, who were eligible for inclusion in the study, of whom 12 were excluded (7 in alfentanil group and 5 in fentanyl group) for poor bowel preparation. A total of 260 patients who had a complete examination were considered in the results of the study, 133 male and 127 female. The baseline characteristics of patients were exactly the same in the two groups (Table 1). About 93.4% of the patients (243/260) were classified as ASA class I or II.

**Table 1:** Basic characteristics of the study groups

**Characteristic**	**Alfentanil group (n=129)**	**Fentanyl group (n=131)**	**P value**
Age, mean±SD (range), y	53.54±10.30 (27–85)	52.34±11.55 (29–87)	0.378
Male/Female	66/63	67/64	1.000
Body weight, mean (SD), kg	62.57 (11.03)	61.55 (11.69)	0.362
BMI, mean (SD), kg/m2	23.86 (3.42)	24.00 (3.75)	0.507
ASA classification, n (%)			1.000
I	39 (30.2)	40 (30.5)	
II	82 (63.6)	82 (62.6)	
III	8 (6.2)	9 (6.9)	
IV	0	0	
Baseline SBP, mean (SD), mm Hg	118.26 (14.27)	121.79 (16.84)	0.070
Baseline DBP, mean (SD), mm Hg	76.11 (10.92)	76.95 (12.83)	0.568
Baseline MAP, mean (SD), mm Hg	89.26 (11.22)	90.98 (13.25)	0.260
Baseline HR, mean (SD), beats/min	71.36 (11.34)	72.65 (11.84)	0.372
Baseline SpO2, mean (SD), %	97.76 (1.02)	97.74 (0.99)	0.874

ASA, American Society of Anesthesiologists; BMI, body mass index; SBP, systolic blood pressure; DBP, diastolic blood pressure.

### Mean total dose of drugs used, total anesthetic cost and procedure- related times

A summary of the mean total dose of sedatives and analgesics administered, total anesthetic cost and the mean procedure-related times are presented in Table 2. No statistically difference was found in any parameter except the total anesthetic cost.

**Table 2:** Sedative analgesic doses, cost and procedure-related times

	**Alfentanil group (n=129)**	**Fentanyl group (n=131)**	**P value**
Total dose, mean±SD (range), mg			
Midazolam	2.5	2.5	
Alfentanil	0.5		
Fentanyl		0.05	
Propofol	108.91±29.19 (20–200)	112.86±31.40 (30–230)	0.973
Total anesthetic cost, mean±SD (range), NT$			
	154.00±9.34 (126–183)	52.77±10.05 (26–90)	<0.001
Procedure-related times, mean±SD (range), min			
Total procedure time	14.97±4.20 (8.10-30.36)	14.90±3.76 (8.02-30.43)	0.331
Awake time	6.35±3.01 (1.10-19.05 )	6.31±2.78 ( 0.82-16.80)	0.899
Recovery time	13.67±4.52 (5.45-30.65)	13.38±3.86 (6.07-28.57)	0.101

The wholesale prices of alfentanil (2 cc ampoule, 0.5 mg/ml) and fentanyl (2 cc ampoule, 0.05 mg/ml) are NT$225 and NT$20, respectively. Each 2 cc ampoule of alfentanil or fentanyl was subdivided for two patients. Therefore, alfentanil 0.5 mg and fentanyl 0.05 mg charged NT$ 113 and NT$10, respectively. Thus, the cost difference was about NT$103, being approximately US$ 4 and leading to a significant difference between the groups.

### Safety profiles

The overall rate of cardiopulmonary adverse events was 10.9% (14/129) in the alfentanil group and 10.7% (14/131) in the fentanyl group (Table 3). However, there was no significant difference between the 2 groups (P=0.966). All complications were successfully managed with conservative care.

**Table 3:** Sedation-associated safety profile, n (%)

	**Alfentanil group (n=129)**	**Fentanyl group (n=131)**	**P value**
Cardiopulmonary complications	14 (10.9)	14 (10.7)	0.966
Systolic hypotension (<90mmHg)	12 (9.3)	11 (8.4)	0.797
MAP decreased >25%	4 (3.1)	2 (1.5)	0.445
Use of vasopressors	1 (0.8)	2 (1.5)	0.445
Bradycardia (<50 beats/min)	0	0	0.445
Hypoxemia (SpO2 < 90%)	4 (3.1)	3 (2.3)	0.721
Chin lift	4 (3.1)	1 (0.8)	0.212

Systolic hypotension was the most frequently reported adverse event in both groups (9.3% [12/129] in the alfentanil group vs 8.4% [11/131] in the fentanyl group; P=0.797). Among these systolic hypotension patients, mean arterial pressure decreased more than 25% of their baseline was 3.1% [4/129] in the alfentanil group vs 1.5% [2/131] in the fentanyl group (P=0.445).

Hypotension was corrected by increasing saline infusion rate and only one patient needed small dose of vasopressor (ephedrine 5 mg IV).

Hypoxemia was the second common adverse event in both groups (3.1% [4/129] in the alfentanil group vs 2.3% [3/131] in the fentanyl group; P=0.721). All patients with hypoxemia required chin lift support only and no assisted ventilation was needed.

One case of bradycardia was noted in fentanyl group and treated with atropine sulfate 0.5 mg. No serious adverse events including the use of flumazenil, permanent injury or death, and temporary or permanent interruption of procedure were found in both groups.

### Patient assessment and satisfaction with sedation

Patient’s sensation of the endoscopic experience is shown in Table 4.

**Table 4:** Patient satisfaction surveys

	**Alfentanil group (n=129)**	**Fentanyl group (n=131)**	**P value**
**Discharge for home**			0.489
**How much pain during the procedure, mean(SD), VAS**			
	0.22 (0.61)	0.17 (0.53)	
**How was the overall satisfaction with the procedure, mean(SD), VAS,**			0.469
	9.43 (0.63)	9.49 (0.57)	
**Do you remember being awake during the procedure? n (%)**			1.000
Yes	1 (0.8)	2 (1.5)	
No	128 (99.2)	129 (98.5)	
**24 hours after procedure**			
**On a scale of 1–10, how would you rate your procedure? mean(SD)**			0.368
	9.76 (0.57)	9.69 (0.59)	
**How was the sedation for your procedure? n (%)**			0.911
Excellent	108 (83.7)	109 (83.2)	
Good	21 (16.3)	22 (16.8)	
Fair	0	0	
Poor	0	0	
**Do you think you needed any adjustment of your sedation? n (%)**			0.245
Needed more	1 (0.8)	0	
Just right	127 (98.4)	131 (100)	
Needed less	1 (0.8)	0	
**Do you remember the start of the procedure? n (%)**			0.445
Yes	4 (3.1)	2 (1.5)	
No	125 (96.9)	129 (98.5)	
**Do you think that the rest of the day was impaired by the sedation? n (%)**			1.000
None	125 (96.9)	126 (96.2)	
Mild	4 (3.1)	5 (3.8)	
Moderate	0	0	
Severe	0	0	
**Did you require additional sleep during the daytime after the procedure? n (%)**			0.374
No	122 (94.6)	127 (96.9)	
Yes	7 (5.4)	4 (3.1)	
Time, mean±SD (range), hour	2.29±1.07 =(1-4)	3.00±1.15 (2-4)	0.351

The immediate post-procedure questionnaire (e.g. pain during the procedure (VAS score), the overall satisfaction with the procedure (VAS score) and being awake during the procedure) were completed by all patients, 129 and 131 patients in alfentanil and fentanyl group, respectively. No difference was found between the groups. The VAS pain score during the procedure was minimal, 0.22±0.61 in the alfentanil group vs 0.17±0.53 in the fentanyl group, (P=0.489). Also, the overall satisfaction with the procedure (VAS score) was high, 9.43±0.63 in the alfentanil group vs 9.49±0.57 in the fentanyl group, (P=0.469). The incidence of being awake during the procedure was low, only 0.8% in the alfentanil group vs 1.5% in the fentanyl group, (P=1).

The survey conducted 24 hours after discharge was also completed by all patients. Similarly, there was no statistically difference between the groups. The overall satisfaction with the sedation was high. 83.7 percent of patients described it as “excellent” and 16.3% rated it “good” in the alfentanil group vs 83.2% described it as “excellent” and 16.8% rated it “good” in the fentanyl group, (P=0.911).

Most patients’ normal activities of the rest of the day were not impaired by the sedation, 96.9% in the alfentanil group vs 96.2% in the fentanyl group, (P=1). The majority of the patients did not require additional sleep after discharge from the endoscopy unit, 94.6% in the alfentanil group vs 96.9% in the fentanyl group, (P=0.374). Few slept for periods of time that ranged from 1 to 4 hours, 2.29±1.07 hour in the alfentanil group vs 3.00±1.15 hour in the fentanyl group, (P=0.351).

## Discussion

This study is the first randomized, double blind controlled trial to compare the efficacy and the safety of small dose alfentanil and fentanyl in BPS titrated to deep sedation for health examination patients undergoing diagnostic endoscopy. Though alfentanil is the preferred narcotic due to its shorter duration and fewer side effects compared with fentanyl; in the present study, there were no differences in the recovery, awake and total procedure times between the groups.

Small doses of alfentanil (incremental dose 0.25 mg, total 0.5 mg) and fentanyl (incremental dose 0.025 mg, total 0.05 mg), were used in the present study might be the main reason.

From Coda’s opinion, fentanyl administrated as a single intravenous dose for short surgical procedure in adults developed as a short-acting opioid and incremental doses of 0.025 to 0.05 mg are recommended [17].

Similarly, the recommended bolus doses of alfentanil are 0.25 to 0.5 mg.

In Usta’s study [11], patient controlled analgesia and sedation with alfentanil or fentanyl combined with midazolam for colonoscopy showed that total sedation times were shorter in the alfentanil group compared with the fentanyl group. The mean dose of alfentanil and fentanyl were 1 mg and 0.08 mg respectively, which was larger than the present study.

In an earlier study, Holloway et al. [18] showed that patients undergoing conscious sedation for colonoscopy with midazolam and alfentanil had better operating conditions compared with midazolam and fentanyl, but the recovery time was similar. The mean doses of alfentanil and fentanyl were low, 0.541 mg and 0.064 mg respectively.

Our narcotic doses and the comparison of recovery time are similar to Holloway's study. It suggests that small dose of narcotics may not influence the recovery time in BPS. Further study with the moderate dose in this issue is warranted.

Small dose of midazolam 2.5 mg (average 0.04 mg/kg) was chosen in the present randomized study. Hayee et al. [19] reported a linear relationship between recovery time and midazolam dose. At lower doses of midazolam (2–3.5 mg), in combination with fentanyl for colonoscopy, mean recovery time was significantly shorter than at higher doses (4-5 mg). The slow metabolization of benzodiazepines could prolong the recovery time, reducing the turnover rate and efficiency of an endoscopic unit. The mean recovery time in the present study was around 15 min which was similar to VanNatta’s study [3]. They showed that their mean recovery time (the time from the end of the procedure to patient discharge) was around 15 minutes for the BPS group targeted to moderate sedation for colonoscopy. However, their midazolam dose was only 1.0 mg and might add advantages to recovery time. Therefore, reduced the midazolam dose in our study might further decrease the recovery time. However, we did not test that hypothesis in this study.

Both types of BPS seem to be equally safe in the present study.

Though deep sedation was targeted, all cardiopulmonary complications were minor and transient. No patient required assisted ventilation or had neurological injury or death. The reasons for the favorable safety profile of this study are as follows. First, it allows precise titration of propofol, with a smaller bolus of doses to maintain an acceptable status of deep sedation. Second, it is clear that a synergistic effect of propofol with benzodiazepines and opioids can reduce the total dose of propofol, thus reducing the severity of cardiopulmonary complications [1-3]. Third, specific antagonists of benzodiazepines (flumazenil) and opioids (naloxone) are available, though no antagonist was required in the present study.

In addition, the incidence of cardiopulmonary complications was increased when propofol alone titrated to deep sedation [20-22]. However, despite numerous publications describing propofol use in endoscopy, there are limited data studying the incidence of cardiopulmonary complications of BPS targeted to deep sedation.

A recent prospective study by Lee et al. [5], in which BPS was targeted to moderate sedation for advanced endoscopic procedures, stated that the incidence of cardiopulmonary complications was 7.2% (15/206) which was lower than in the present study (10.8%, 28/260).

Though not directly compare, it seems that BPS targeted to deep sedation might have higher cardiopulmonary complications than titrated to moderate sedation. Further direct compared study of this issue is warranted.

Interestingly, a recent study by Agostoni et al. [23] showed that deep sedation was achieved with propofol monotherapy with a target controlled infusion (TCI) and the incidence of adverse events was very low, only 5.88% in combined endoscopic procedures. The TCI technique decreasing the amount of infused drugs might be the reason. Direct compared study between BPS and TCI technique is warranted.

Furthermore, hypoxemia (SpO_2_ <90%) is one of the major cardiopulmonary complications of propofol based sedation. Coté et al. [24] stated that despite supplemental oxygen by nasal cannula (2 L/min), when most of the patients (87.2%) met the criteria for deep sedation during advanced endoscopic procedures, hypoxemia occurred in 12.8%, which was much higher than the present study (2.6%). Preoxygenation with intranasal oxygen (6 L/min) providing to all patients in the present study might be the key factor of the difference.

A recent retrospective study found that under supplemental oxygen (4 L/min) through a nasal cannula, hypoxemia was only 0.6% during colonoscopy in deeply sedated patients [12]. Similarly, under 4 L/min intranasal oxygen supplement, hypoxemia was not happened when BPS titrated to moderate sedation for colonoscopy [3].

In addition, at the emergency department, Deitch et al. reported that compared with compressed air, high-flow oxygen 15 L per minute by a non-rebreathed mask [25], but not nasal cannula oxygen at 2 L/min [26], reduces the frequency of hypoxia during propofol sedation in adults.

Thus, it suggests that intranasal preoxygenation with high-flow oxygen 4 to 6 L per minute should be routinely administered to patient undergoing propofol based sedation targeted to deep sedation for diagnostic endoscopy.

However, supplemental oxygen can delay the occurrence of hypoxemia, provide a false sense of security, and thus delay in diagnosis of respiratory depression and airway obstruction. Therefore, it is recommended that monitoring of ventilatory function should also include close patient observation as in the present study.

The other important finding in this study was the high patient overall satisfaction with the procedure. While discharge for home, the VAS score of overall satisfaction with the procedure was up to 9.43 in the alfentanil group and 9.49 in the fentanyl group. Re-assessed 24 hours after the procedure again confirmed a high level of patient satisfaction; the 10 point scale was up to 9.76 in the alfentanil group and 9.69 in the fentanyl group.

Deep sedation, causing the patient to be more comfortable, which was chosen in the present study, might be the key factor. Actually, in Van Natta’s study, propofol alone titrated to deep sedation had higher patient satisfaction than propofol in combination with opioids and benzodiazepines targeted to moderate sedation for colonoscopy, though no statistical significance was found [3].

Though at present, there is no evidence supporting that deep sedation might increase patient satisfaction; additional study of this issue is warranted, especially whether there is any difference between deep and moderate sedation of BPS. Deep sedation might also achieve better colon cancer discovery rate [27,28] and higher level of endoscopist satisfaction [29].

Moreover, sedation technique might also influence the patient overall satisfaction. Fanti et al. [30] reported that patient-controlled analgesia targeted to moderate sedation also achieved a high patient overall satisfaction up to 9.11 VAS score. Further direct compared study among different sedation techniques is necessary.

Some would argue whether deep sedation is really desirable for healthy patients with diagnostic endoscopic procedure. Internationally, sedation for colonoscopy varied widely and nearly 30% colonoscopic procedure received deep sedation [31]. In Taiwan, most of patients and endoscopists favored deep sedation for the endoscopic procedures despite greater use of resource, monitoring and anesthetist [12,13]. Further worldwide observation is warranted.

Furthermore, the mean total procedure time was 15 minutes in the present study. It appeared rather short for EGD and colonoscopy and in the light of the recommended 6 minutes withdrawal time. A skillful gastroenterologist with more than 30,000 endoscopic and 15 years experiences was involved in the study might be the key reason of the short total procedure time. The mean withdrawal time was 3.94 minutes in the present study.

With regard to the individual patient's anesthetic costs, including all the anesthetic drugs consumed only, showed a significant difference between groups. Since the doses of midazolam and propofol are not different between groups, the cost of alfentanil and fentanyl is the key factor of the marked difference. Actually, due to no generic product of alfentanil available at present time, alfentanil 0.5 mg is about 11 times expensive than fentanyl 0.05 mg, which is not offset by other costs such as decreased operation time, shorter recovery time or hospital stay, and earlier resumption of normal activities. Therefore, the use of alfentanil, added about US$ 4 per patient to the procedure costs, could be a significant institutional financial issue.

Moreover, since BPS is administrated safely by non-anesthetists in many countries, and there is no evidence that the very costly presence of an anesthetist improves the patient safety. Non-anesthetist delivered sedation should become dominant in the world. However, it is surprising that anesthesia professional-delivered sedation for EGD and colonoscopy has become more common in the United States. From 2003 to 2007, the involvement of anesthesiologists in colonoscopy increased from 9 to 25%, and this might be up to more than 50% by 2015 [32]. Further observation is warranted.

There are limitations of our study. First, this study was performed in a center with only one gastroenterologist and anesthesiologist, and followed up by an independent research nurse. Therefore, the results are difficult to generalize. Multiple-center clinical trial might solve this limitation.

In addition, individual differences in clinical assessment and management may exist among anesthesiologists, gastroenterologists and also research nurses, resulting in information bias and failing to reject the null hypothesis of the study. Thus, individual difference would be avoided in the present study. Second, the potency ratio for fentanyl:alfentanil of 10:1 was chosen because of the convenient to calculate in a clinical setting. However, this ratio is not equipotent. According to Stanski et al., alfentanil is five to six times less potent than fentanyl [33]. Moreover, the opioid dosage may also have been influenced by the amount of propofol administered to maintain a constant level of sedation. Because there is no difference in total propofol consumption and also the procedure time between the groups, it suggests that the convenient ratio is adequate in the present study.

## Conclusion

In conclusion, the findings of the present sedation regimens demonstrate that small doses of alfentanil and fentanyl are equally effective adjuncts to BPS titrated to deep sedation for EGD and colonoscopy in health examination patients. No difference in the propofol consumption, awake time from sedation, recovery time, sedation-related cardiopulmonary complications and satisfaction score from patients was found between the two groups. The only practical difference appears to be the cost because of the great difference in price between alfentanil and fentanyl. This evaluation of sedation regimens may be useful for the future development of new BPS protocols to reduce the cost to a minimum.

## Appendix

24-hour telephone questionnaire Q1: On a scale of 1–10, how would you rate your procedure? Q2: How was the sedation for your procedure? Excellent Good Fair Poor Q3: Do you think you needed any adjustment of your sedation?Needed more Just right Needed less Q4: Do you remember the start of the procedure? Yes No Q5: Do you remember being awake during the procedure? Yes No Q6: Do you remember the end of the procedure when the instrument was removed? Yes No Q7: Do you think that the rest of your day was impaired by the sedation?None Mild Moderate Severe Q8. Did you require additional sleep during the daytime after the procedure?No Yes Time (hour) ______

## Abbreviations

BPS: Balanced propofol sedation; EGD: Esophagogastroduodenal endoscopy; NT$: New Taiwan dollar; OAAS: Sale Observer’s assessment of alertness/sedation scale.

## Competing interests

None of the authors has any financial or non-financial competing interests influencing interpretation of data or presentation of information.

## Authors' contributions

WMH and MCC conceived the study. WMH, CMY and CHL designed the study. WMH and CYL conducted of the study. KLH, CMY and CHL were responsible for data analysis. All authors were responsible for manuscript preparation. All authors reviewed and approved the final manuscript.

## Pre-publication history

The pre-publication history for this paper can be accessed here:

http://www.biomedcentral.com/1471-230X/12/164/prepub
